# Supplementary figures and images for: Major chemical carcinogens and health exposure risks in some therapeutic herbal plants in Nigeria
Source: PLoS One. 2022 Nov 3;17(11):e0276365. doi: 10.1371/journal.pone.0276365 (PMC9632904; doi:10.1371/journal.pone.0276365)

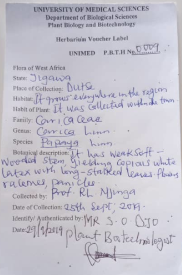

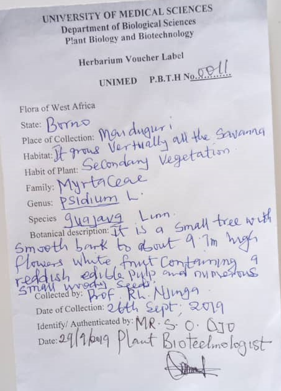


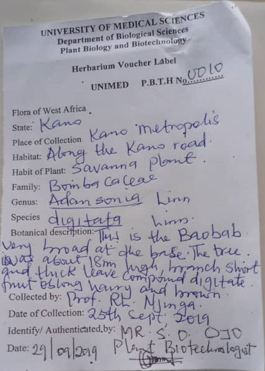

Supplement: S1 Raw images — (DOCX) [file pone.0276365.s001.docx]
