# Supplementary material for: Major chemical carcinogens and health exposure risks in some therapeutic herbal plants in Nigeria
Source: PLoS One. 2022 Nov 3;17(11):e0276365. doi: 10.1371/journal.pone.0276365 (PMC9632904; doi:10.1371/journal.pone.0276365)
Supplement: S1 File — (DOCX) [file pone.0276365.s002.docx]

Dear Editor,

I write to confirm that the submission contains the "minimal data set". All the data set used to reach the conclusions drawn in the manuscript with related metadata and methods, and any additional data required to replicate the reported study findings in their entirety has been included. This include the;

1. values behind the means, standard deviations and other measures reported,
2. values used to build graphs,
3. points extracted for analysis.

All the task requested have been performed. However, feel free to contact us if you need further clarifications.
