# Supplementary material for: Major chemical carcinogens and health exposure risks in some therapeutic herbal plants in Nigeria
Source: PLoS One. 2022 Nov 3;17(11):e0276365. doi: 10.1371/journal.pone.0276365 (PMC9632904; doi:10.1371/journal.pone.0276365)
Supplement: Attachment — Submitted filename: Rebuttal letter 1.docx [file pone.0276365.s003.docx]

MAJOR CHEMICAL CARCINOGENS AND HEALTH EXPOSURE RISK IN SOME THERAPEUTIC HERBAL PLANTS IN NIGERIA

PLOS ONE

Dear Dr. Njinga,

Thank you for submitting your manuscript to PLOS ONE. After careful consideration, we feel that it has merit but does not fully meet PLOS ONE’s publication criteria as it currently stands. Therefore, we invite you to submit a revised version of the manuscript that addresses the points raised during the review process.

Please submit your revised manuscript by Mar 06 2022 11:59PM. If you will need more time than this to complete your revisions, please reply to this message or contact the journal office at [plosone@plos.org](mailto:plosone@plos.org). When you're ready to submit your revision, log on to https://www.editorialmanager.com/pone/ and select the 'Submissions Needing Revision' folder to locate your manuscript file.

Please include the following items when submitting your revised manuscript:

- A rebuttal letter that responds to each point raised by the academic editor and reviewer(s). You should upload this letter as a separate file labeled 'Response to Reviewers'.
- A marked-up copy of your manuscript that highlights changes made to the original version. You should upload this as a separate file labeled 'Revised Manuscript with Track Changes'.
- An unmarked version of your revised paper without tracked changes. You should upload this as a separate file labeled 'Manuscript'.

If you would like to make changes to your financial disclosure, please include your updated statement in your cover letter. Guidelines for resubmitting your figure files are available below the reviewer comments at the end of this letter.

If applicable, we recommend that you deposit your laboratory protocols in protocols.io to enhance the reproducibility of your results. Protocols.io assigns your protocol its own identifier (DOI) so that it can be cited independently in the future. For instructions see: <https://journals.plos.org/plosone/s/submission-guidelines#loc-laboratory-protocols>. Additionally, PLOS ONE offers an option for publishing peer-reviewed Lab Protocol articles, which describe protocols hosted on protocols.io. Read more information on sharing protocols at <https://plos.org/protocols?utm_medium=editorial-email&utm_source=authorletters&utm_campaign=protocols>.

We look forward to receiving your revised manuscript.

Kind regards,

Noshin Ilyas
Academic Editor
PLOS ONE

Journal Requirements:

When submitting your revision, we need you to address these additional requirements.

1. Please ensure that your manuscript meets PLOS ONE's style requirements, including those for file naming. The PLOS ONE style templates can be found at 
https://journals.plos.org/plosone/s/file?id=wjVg/PLOSOne_formatting_sample_main_body.pdf and 
https://journals.plos.org/plosone/s/file?id=ba62/PLOSOne_formatting_sample_title_authors_affiliations.pdf. **Response: Formatted as requested**

2. Thank you for including information about the identification of the samples used in this study. Please provide the name of the expert consulted in identifying the samples. **Response: We have cited the expert with similar medicinal plants in their articles**

Additionally, in your Methods section, please provide additional information regarding the permits you obtained for the work. Please ensure you have included the full name of the authority that approved the field site access and, if no permits were required, a brief statement explaining why. **Response: No permit is needed since this is an individual research work and might be used by the government as baseline data.**

3. Thank you for stating the following financial disclosure:

"No"

At this time, please address the following queries:

a) Please clarify the sources of funding (financial or material support) for your study. **Response: I used my personal salary to fund the research.**
b) List the grants or organizations that supported your study, including funding received from your institution. **Response: No support from anywhere.**
c) State what role the funders took in the study. **Response: No support from anywhere**
d) If the funders had no role in your study, please state: **“The funders had no role in study design, data collection and analysis, decision to publish, or preparation of the manuscript.**

4. In your Data Availability statement, you have not specified where the minimal data set underlying the results described in your manuscript can be found. **Response: Provided as requested**

PLOS defines a study's minimal data set as the underlying data used to reach the conclusions drawn in the manuscript and any additional data required to replicate the reported study findings in their entirety. All PLOS journals require that the minimal data set be made fully available. For more information about our data policy, please see http://journals.plos.org/plosone/s/data-availability.

Upon re-submitting your revised manuscript, please upload your study’s minimal underlying data set as either **Supporting Information files** or to a stable, public repository and include the relevant URLs, DOIs, or accession numbers within your revised cover letter. For a list of acceptable repositories, please see http://journals.plos.org/plosone/s/data-availability#loc-recommended-repositories. Any potentially identifying patient information must be fully anonymized.

Important: If there are ethical or legal restrictions to sharing your data publicly, please explain these restrictions in detail. Please see our guidelines for more information on what we consider unacceptable restrictions to publicly sharing data: http://journals.plos.org/plosone/s/data-availability#loc-unacceptable-data-access-restrictions. Note that it is not acceptable for the authors to be the sole named individuals responsible for ensuring data access.

We will update your Data Availability statement to reflect the information you provide in your cover letter.

5. We note that Figure 1 in your submission contain copyrighted images. **Response: Figure 1 has been removed.**

All PLOS content is published under the Creative Commons Attribution License (CC BY 4.0), which means that the manuscript, images, and Supporting Information files will be freely available online, and any third party is permitted to access, download, copy, distribute, and use these materials in any way, even commercially, with proper attribution. For more information, see our copyright guidelines: http://journals.plos.org/plosone/s/licenses-and-copyright.
 
We require you to either (1) present written permission from the copyright holder to publish these figures specifically under the CC BY 4.0 license, or (2) remove the figures from your submission: **Response: Figure 1 has been removed.**
 
a. You may seek permission from the original copyright holder of Figure 1 to publish the content specifically under the CC BY 4.0 license. **Response: Figure 1 has been removed.**
 
We recommend that you contact the original copyright holder with the Content Permission Form (http://journals.plos.org/plosone/s/file?id=7c09/content-permission-form.pdf) and the following text:
“I request permission for the open-access journal PLOS ONE to publish XXX under the Creative Commons Attribution License (CCAL) CC BY 4.0 (http://creativecommons.org/licenses/by/4.0/). Please be aware that this license allows unrestricted use and distribution, even commercially, by third parties. Please reply and provide explicit written permission to publish XXX under a CC BY license and complete the attached form.” **Response: Figure 1 has been removed.**
 
Please upload the completed Content Permission Form or other proof of granted permissions as an "Other" file with your submission. **Response: Figure 1 has been removed.**
 
In the figure caption of the copyrighted figure, please include the following text: “Reprinted from [ref] under a CC BY license, with permission from [name of publisher], original copyright [original copyright year].” **Response: Figure 1 has been removed.**
 
b. If you are unable to obtain permission from the original copyright holder to publish these figures under the CC BY 4.0 license or if the copyright holder’s requirements are incompatible with the CC BY 4.0 license, please either i) remove the figure or ii) supply a replacement figure that complies with the CC BY 4.0 license. Please check copyright information on all replacement figures and update the figure caption with source information. If applicable, please specify in the figure caption text when a figure is similar but not identical to the original image and is therefore for illustrative purposes only. **Response: Figure 1 has been removed.**

Additional Editor Comments:

This paper needs comprehensive revisions, more details are required about collected samples, statistical analysis and work. **Response: A comprehensive revision has been done as requested and highlighted in red.**

Moreover, authors should add recent literature in introduction and discussion. **Response: More recent literature has been included as requested and the older ones removed.**

Reviewers' comments should also be properly addressed **Response: Done properly**

[Note: HTML markup is below. Please do not edit.]

Reviewers' comments:

Reviewer's Responses to Questions

**Comments to the Author**

1. Is the manuscript technically sound, and do the data support the conclusions?

The manuscript must describe a technically sound piece of scientific research with data that supports the conclusions. Experiments must have been conducted rigorously, with appropriate controls, replication, and sample sizes. The conclusions must be drawn appropriately based on the data presented.

Reviewer #1: Partly

Reviewer #2: Partly

2. Has the statistical analysis been performed appropriately and rigorously?

Reviewer #1: No **Response: We have performed rigorous statistical analysis as requested.**

Reviewer #2: No **Response: We have performed rigorous statistical analysis as requested.**

3. Have the authors made all data underlying the findings in their manuscript fully available?

The [PLOS Data policy](http://www.plosone.org/static/policies.action#sharing) requires authors to make all data underlying the findings described in their manuscript fully available without restriction, with rare exception (please refer to the Data Availability Statement in the manuscript PDF file). The data should be provided as part of the manuscript or its supporting information, or deposited to a public repository. For example, in addition to summary statistics, the data points behind means, medians and variance measures should be available. If there are restrictions on publicly sharing data—e.g. participant privacy or use of data from a third party—those must be specified.

Reviewer #1: Yes

Reviewer #2: No **Response: We have provided the data as requested.**

4. Is the manuscript presented in an intelligible fashion and written in standard English?

PLOS ONE does not copyedit accepted manuscripts, so the language in submitted articles must be clear, correct, and unambiguous. Any typographical or grammatical errors should be corrected at revision, so please note any specific errors here.

Reviewer #1: Yes

Reviewer #2: No. **Response: We have reviewed the entire manuscript.**

5. Review Comments to the Author

Please use the space provided to explain your answers to the questions above. You may also include additional comments for the author, including concerns about dual publication, research ethics, or publication ethics. (Please upload your review as an attachment if it exceeds 20,000 characters)

Reviewer #1: The manuscript entitled "MAJOR CHEMICAL CARCINOGENS AND HEALTH EXPOSURE RISK IN SOME
THERAPEUTIC HERBAL PLANTS IN NIGERIA" can be accepted with major revisions.

Methodology and result section is poorly written in non-technical way. **Response: We have written it properly as requested.**

Results in table 2 reveal that, the amount of Cd in C. guajava collected from Kanu, and Jigwa was .037 and .05 mg/ kg which was little bit higher than WHO standards (.02 mg/kg); further its significance should be tested with z-test. On the other hand the Cr as depicted in table 2, is in safe-limits (less than 1.3 mg/kg); its significance can also be test by z-test. **Response: Statistical analysis has been performed to establish this fact.**

In table 3, the EDI is also within the acceptable limits, so what is the author claiming for? **Response: The research work has to show this fact as a baseline data.**

Further more, I would like to recommend some statistical analyses to make the manuscript findings better understandable. **Response: Statistical analysis has been performed.**

There would be analysis of variance (ANOVA) using two factors (Locations and Plant-species) using HSD as post-hoc for each studied variable. **Response: Statistical analysis of variance (ANOVA) using two factors (Locations and Plant-species) using HSD as post-hoc for each studied variable has been done.**

Strictly, there would be a z-test for EDI, THQ, HI, TCR in which author should compare the observed average values with standard acceptable values; the results would be presented in the form of z-values, and p-values for each variable. Without the z-test, the findings and claim of the author is NOT reliable. **Response: Statistical t-test for EDI, THQ, HI, TCR has been perform since the data is not large enough for z-test.**

Quality of graphs (data visualization) is poor can be made better by highlighting (horizontal line at y-axis parallel to x-axis) the acceptable ranges in each graph. **Response: The lines are very good as it is as compared to other article published.**

Reviewer #2: Dear Authors,

The article, “MAJOR CHEMICAL CARCINOGENS AND HEALTH EXPOSURE RISK IN SOME THERAPEUTIC HERBAL PLANTS IN NIGERIA” highlights the issue contamination in herbal remedies. The heavy metals (Cd and Cr) are the major toxicants in plants (Herbs or Vegetables), and these metals are sourced from irrigation water or the polluted soil. However, as I reviewer I have some comments that need to be addressed before reaching any final decision.

First the abstract has to be revised thoroughly **Response: It has been revised thoroughly**, the second sentence starting “Accumulation of MCCs….” Is confusing and need to be revised**. Response: It has been revised thoroughly**. I am wondering how the authors named heavy metals (Cd and Cr) as MCC?? **Response: This has been well classified as MCC (Arsenic, cadmium, chromium, and nickel are classified as group 1 carcinogens by the International Agency for Research on Cancer, and are utilized commercially).**

Abstract has many typos, difficult to highlight each one here. It’s not clear which plant has higher concentration of these metals? **Response: It has been revised thoroughly**

Why the authors have not considered investigating several other heavy metals? I wonder if the authors have also studied the nutritionally important metals in these plants? **Response: We are working on other heavy metals considering other matrices and will be submitting for possibly publication. About the nutritional importance, we shall focus on that after. Thanks for the suggestion.**

The introduction part is weak, it needs improvement in terms of detailed information of C. papaya, P. guajava, and A. digitate. **Response: This will make the paper very lengthy. However, we have revised the introductory part thoroughly.** The introduction section just lists the review of literature it lacks the real on-ground information, socio-economic value of these plants? Also, It will be good to have pictures of these plants and the plant distribution in Nigeria, etc. **Response: It has been revised thoroughly**

The presented data lack the proper statistical analysis. **Response: We have included the statistical analysis.**  As per my understanding, the plant samples were collected from different locations in Nigeria, **Response: They were collected from three states as described.** Hence it will be best to point to these localities with a higher concentration of heavy metals in samples. **Response: These samples were collected randomly without GPS.**  This information will be good for the folks and herbalists to refrain to use these plants in their formulations. **Response: The new work we are currently working on will carry all these suggestions as we will return to the field to get all the GPS of the locations and map them. However, these values were recorded in average from each of the states.**

The number of samples per plant sample is low I just wonder if the toxic metals are really sourced from plants or in solvents?? **Response: The number of samples per plants was based on initial results. They were almost similar per state. The heavy metal was analysis using AAS as described in the methodology section of the work.** Did the Authors try to analyze these metals in the solvents? **Response: No**

6. PLOS authors have the option to publish the peer review history of their article ([what does this mean?](https://journals.plos.org/plosone/s/editorial-and-peer-review-process#loc-peer-review-history)). If published, this will include your full peer review and any attached files.

If you choose “no”, your identity will remain anonymous but your review may still be made public.

**Do you want your identity to be public for this peer review?** For information about this choice, including consent withdrawal, please see our [Privacy Policy](https://www.plos.org/privacy-policy).

Reviewer #1: No

Reviewer #2: No

[NOTE: If reviewer comments were submitted as an attachment file, they will be attached to this email and accessible via the submission site. Please log into your account, locate the manuscript record, and check for the action link "View Attachments". If this link does not appear, there are no attachment files.]

While revising your submission, please upload your figure files to the Preflight Analysis and Conversion Engine (PACE) digital diagnostic tool, <https://pacev2.apexcovantage.com/>. PACE helps ensure that figures meet PLOS requirements. To use PACE, you must first register as a user. Registration is free. Then, login and navigate to the UPLOAD tab, where you will find detailed instructions on how to use the tool. If you encounter any issues or have any questions when using PACE, please email PLOS at [figures@plos.org](mailto:figures@plos.org). Please note that Supporting Information files do not need this step.
