# Supplementary material for: Major chemical carcinogens and health exposure risks in some therapeutic herbal plants in Nigeria
Source: PLoS One. 2022 Nov 3;17(11):e0276365. doi: 10.1371/journal.pone.0276365 (PMC9632904; doi:10.1371/journal.pone.0276365)
Supplement: Attachment — Submitted filename: Response to Reviewer.docx [file pone.0276365.s004.docx]

PLOS ONE: Edits requested on your submission PONE-D-21-39473R2 - [EMID:799aef60686c7ddd]

rnjinga/Inbox

-

**PLOS ONE** <em@editorialmanager.com>

**To:**RAYMOND LIMEN Njinga

Fri, 30 Sept at 01:34

=============================================

IMPORTANT: PLEASE DO NOT REPLY TO THIS EMAIL

If you are unable to complete any points that are requested in this email, please explain why in the 'Enter Comments' tab of the online submission form prior to re-submitting your manuscript. This will let us quickly assess your response and send your manuscript to an Academic Editor as soon as possible.

=============================================

PONE-D-21-39473R2

MAJOR CHEMICAL CARCINOGENS AND HEALTH EXPOSURE RISK IN SOME THERAPEUTIC HERBAL PLANTS IN NIGERIA

Prof RAYMOND LIMEN Njinga

Dear Dr. Njinga,

We've checked your submission and before we can proceed, we need you to address the following issues:

1. We note that several of your files are duplicated on your submission. Please remove any unnecessary or old files from your revision, and make sure that only those relevant to the current version of the manuscript are included.

**Response: Done**

2. Please amend the title either on the online submission form or in your manuscript so that they are identical. **Response: Done**

3. Please include a copy of Table 5 which you refer to in your text on page 13. Or if the table is no longer to be included as part of the submission please remove all reference to it within the text.

**Response: Done**

4. Please ensure that you refer to Table 6 in your text as, if accepted, production will need this reference to link the reader to the Table.

**Response: Table 6 has been changed to Table 5.**
